# Data integration of electronic medical record under administrative decentralization of medical insurance and healthcare in China: a case study

**DOI:** 10.1186/s13584-019-0293-9

**Published:** 2019-04-01

**Authors:** Zhong Wang

**Affiliations:** Economic Institute, Beijing Academy of Social Sciences, No. 33, North Fourth Ring Road, Chaoyang District, Beijing, 100101 China

**Keywords:** Electronic medical record, Data integration, Medical insurance administration, Healthcare administration, Administration institutions

## Abstract

In most regions of China, Electronic Medical Record (EMR) systems in hospitals are developed in an uncoordinated manner. Medical Insurance and Healthcare Administration are localised and organizations gather data from a functional management viewpoint without consideration of wider information sharing. Discontinuity of data resources is serious. Despite the government’s repeated emphasis on EMR data integration, little progress has been made, causing inconvenience to patients, but also significantly hindering data mining.

This exploratory investigation used a case study to identify bottlenecks of data integration and proposes countermeasures. Interviews were carried out with 27 practitioners from central and provincial governments, hospitals, and related enterprises in China. This research shows that EMR data collection without patients’ authorization poses a major hazard to data integration. In addition, non-uniform information standards and hospitals’ unwillingness to share data are also significant obstacles to integration. Moreover, friction caused by the administrative decentralization, as well as unsustainability of public finance investment, also hinders the integration of data resources.

To solve these problems, first, a protocol should be adopted for multi-stakeholder participation in data collection. Administrative authorities should then co-establish information standards and a data audit mechanism. Finally, measures are proposed for expanding data integration for multiplying effectiveness and adopting the Public-Private Partnerships model.

## Introduction

In recent years, the Electronic Medical Record (EMR) has become an important source for big data analysis, beyond its traditional use in disease treatment [1]. For example, it can be used to drive decision-making in public health programmes [2], identify risk factors for infectious diseases [3], enable continuity of care between medical institutions [4], improve healthcare quality, facilitate medical research [5], enhance epidemiological surveillance and reporting, support clinical decisions [6] and so on. All this highlights the growing recognition of the immense value of EMR data and the increasing expectations for its use. Nevertheless, in order to use this material to its full, it is an essential prerequisite that the data is integrated [7]. Data mining to yield greater knowledge and provide valuable research insights requires the compatibility of EMR data from multiple hospitals [8]. Actually, integration of healthcare data from numerous providers within a region or a country has become a practical necessity. Great efforts have been made by many countries, such as Germany [9], the UK [10] and others, to achieve just this.

China is likely to be the country with the largest volume of EMR data because of its vast population and the rapid development and deployment of information systems across the country. However, unfortunately at present, if patients change hospital, the receiving doctor cannot usually access all their records from previous hospitals. When they go to other provinces for medical treatment, medical insurance (MI) cannot reimburse the expenses incurred. The crux of these problems lies in the lack of data integration. In June 2016, in order to promote data integration and utilization, China’s State Council issued Opinions on Promoting and Regulating the Development of Big Data Applications in Healthcar*e*. It aims to accelerate the construction of a unified population healthcare information platform including four levels: national, provincial, municipal, and county. Taking electronic medical records as the core resource, the platform will build healthcare big data resources and promote the sharing of these resources. Despite the release of these regulations and financial support from all levels of the government, little progress has yet been made in EMR data integration.

What are the bottlenecks that EMR data integration has encountered in China? What countermeasures should be taken? In order to learn about these issues, we conducted an exploratory study by interviewing 27 typical stakeholders from governments, hospitals, and related enterprises. Based on these interviews, this paper studies the bottlenecks in integrating EMR data and then makes suggestions for addressing the barriers identified.

## Background

**Fig. 1:**
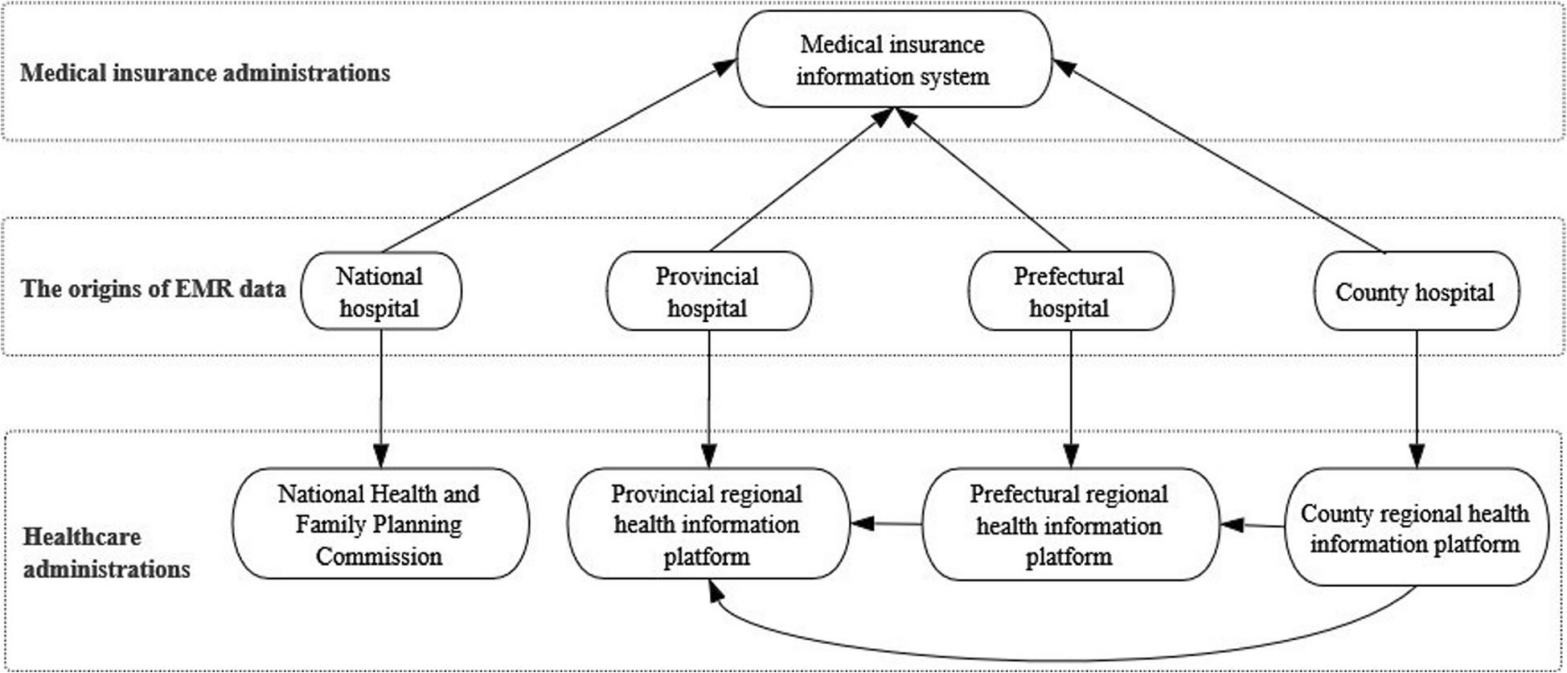
The relationship of data collectors. The hospital is the original data collector. MIA and HA gather data separately from hospitals, whose information platforms are MIIS and RHIP respectively

After putting in place the basic hardware and software infrastructure for effective data capture, the Chinese government gave increasing attention to healthcare data available in an effort to use this valuable resource. For the sake of cost control, Healthcare and Medical Insurance Administration are decentralized in China. Medical Insurance Administration (MIA) mainly formulates medical insurance systems, constructs and implements the supervision and management of medical insurance funds, and produces medical insurance catalogues and payment standards for medicines and medical service. Healthcare Administration (HA) is mainly responsible for allocating the medical and health service resources, supervising and managing public health and medical services, as well as constructing the population health information platform. MIA and HA gather data separately from hospitals to fulfil their management functions. Their information platforms are the Medical Insurance Information System (MIIS) and the Regional Health Information Platform (RHIP, which is also referred to as the “Regional Population Health Information Platform” in some provinces) (Fig. 1).

### Hospital and EMR system

China’s hospital administration system is complicated and bureaucratic. Public hospitals have administrative hierarchies, which are principally divided into four levels. (1) National hospitals. There are 44 hospitals in total governed by the central government, specifically the National Health and Family Planning Commission. (2) Provincial hospitals. The governing authority of these hospitals is the HA of the provinces, municipalities or autonomous regions. (3) Prefectural hospitals. These hospitals are under the management of the prefectural HA. (4) County hospitals. In general, the local HA is the governing authority. In addition, there are some military hospitals, which have an independent management system.

Patients often go directly to hospitals rather than community health services due to their distrust of the latter’s medical expertise and technology. Unlike the UK, hospitals do not require a referral from a primary care surgery. In China, community surgeries mainly undertake the functions of vaccination and some auxiliary work for hospitals, such as injections, dressing changes and similar routine procedures. Less than one-third of the 2600 existing surgeries are financially viable in a densely populated city like Beijing. Most of them need subsidies to maintain operations. Due to insufficient funds and poorly qualified personnel, the quality of data collected in the community healthcare services is poor. Moreover, some of the data has been uploaded to hospitals because some community healthcare services are branches of hospitals. Therefore, it is most economical to integrate EMR data from hospitals. However, in most regions in China, EMR systems usually evolve independently, which leads to the development of information silos.

### HA and RHIP

Although HA has been renamed several times (the current name National Health Commission in March 2018), one of its important functions of RHIP construction, has not been changed. RHIP collects EMR data from its subordinate hospitals. Some provincial RHIPs also collect data from prefectural and county RHIPs. According to the plan of the central government, requirements for RHIP construction include unified standards, openness, hierarchical administration, safety and reliability. At present, in response to this ambitious vision, some provincial governments have completed initial phases of RHIP construction. For example, as one of the more developed provinces, Jiangsu has collected and integrated EMR data of 80 million citizens from most hospitals in the province. However, most provinces are just starting their work on RHIP, and some have not even finished the data centralization at the county level.

### MIA and MIIS

Compared to HA’s complex structure, MIA looks simpler, as depicted in Fig. 1. Actually, the simple structure is only for the urban population (including urban employees and urban residents). The MIIS for urban people is unified at the provincial level and national networking was completed in September 2017. However, for rural people, the data integration situation is the same as HA, because their medical insurance fund was established and managed by HA. It was not until 2015 that the government decided to centralize medical insurance for urban and rural residents. Both MIA and HA wanted to obtain the administrative authority and rights over this merger. After fierce competition with HA, MIA won.

At present, the number of MI participants exceeds 1.3 billion and universal coverage has basically been achieved. Almost all the hospitals are MIA-designated hospitals, except for a small number of expensive private hospitals, and the new hospitals during the inspection period (their first year). However, the MIIS for rural people differs from province to province. A number of these systems are provincially standardized, while others are unified at the prefecture level or county level. Data integration work is still in progress.

## Literature review

As an important resource, the collection and integration of EMR data have become a research hotspot for many years. The literature related to this study includes the following:

1. Collection of EMR data.
  Mursaleen [11] surveyed the attitudes of patients with Parkinson’s disease towards data collection and found that 93% of respondents were willing to share data, yet only 41% were currently doing so. Many parties would like easy access to EMR in pursuit of a good cause, whilst those advocating a high privacy threshold do not support broadbased access due to the risk of breaching medical confidentiality [12]. While some people are committed to pursuing a balanced approach between these conflicting interests, others are more willing to sacrifice one side of the argument in favour of their preferred option. [13]. Although people have different ideas, it is widely accepted that legislation should ensure the anonymity of the patient and encourage participation in the health delivery system and in scientific research [14], as well as support public registries in collecting and using health data for the public interest without overriding the consent of data subjects [15].
2. Integration of EMR data.
  The effective integration of EMR data from lots of hospitals is a prerequisite for big data analysis. Many countries and regions are putting great efforts into this field. For example, the Health and Social Care Information Centre in England collects medical records data from public medical institutions and GPs and integrates them in a national database [10]. Germany released the Medical Informatics Initiative to improve the possibilities for medical research and patient care through innovative IT solutions [9]. In 2010, the Chinese government issued the Basic Standards for Electronic Medical Records (Trial). Since then, thousands of EMR systems have been developed by information technology service enterprises [16]. Many local governments are exploring the integration of EMR data, possibly through a medical cloud storage information platform, which they hope will improve the accuracy of diagnosis, increase appropriate treatment levels of primary hospitals and at the same time will reduce patient medical costs [17]. The previous studies found that the main problems were the absence of a uniform information standard, security problems, shortage of funds and lack of qualified administrators, and suggested that the health administration should formulate a commonly applied standard, strengthen data security, increase public financial investment and implement human resources training [18].

The studies mentioned above mainly analyzed China’s EMR data integration from the perspective of information technology and HA, thus lacked in-depth institutional analysis of the EMR data integration. In particular, they neglected to take account of MIA, which has significant influence on hospital dynamics. Conducting research in these areas of deficiency will contribute to healthcare information development in China, as well as provide a reference for other countries. Therefore, this article mainly studies the bottlenecks of integrating EMR data under the current administrative decentralization in China, and also puts forward several suggestions of a way forward.

## Methodology

In order to understand EMR data integration policy, the present situation and problems, the project team conducted interviews with typical stakeholders from October 2016 to April 2017. It was not feasible to conduct a large-scale survey because making appointments with interviewees was very difficult. Therefore, the case study method was adopted. The interview sample was limited by the availability of informants, given their workload or willingness. Table 1 provides information on the types of people interviewed. Due to the respondent’s request for anonymity, a detailed list will not be provided here.

**Table 1:** Overview of interviewees

Organization type	Role of interviewees	Types of interviews	The number of interviewees
Central government	Officials in National Health and Family Planning Commission.	group interview	2
	Officials in Ministry of Human Resources and Social Security of China.	group interview	2
Provincial governments	Leaders of the MIA and HA in A.	2 individual interviews	2
	Head of information department in HA in B.	individual interview	1
	Head of information department in HA in C.	individual interview	1
Hospitals	Persons in charge of health informatics from five well-known hospitals in Beijing.	5 individual interviews	5
Companies	Heads of five health information technology enterprises.	group interview	7
	Leaders from two MI information technology enterprises.		3
	Leaders from two commercial health insurance companies.	group interview	4
Total			27

First, we interviewed two officials in each of the two ministries of the Central Government: National Health and Family Planning Commission and Ministry of Human Resources and Social Security of China. All respondents are from the information management department of the ministries. An open-ended interview was held for about 60 min with 2 respondents at each ministry. The contents of the interviews were mainly about the status quo, problems, prospects and suggestions on EMR data integration.

Secondly, since the development levels of EMR data integration vary among the provincial administrative regions, three of the more developed regions were selected from all 34 regions, using A, B, and C as aliases. Two interviews were conducted in A, targeting separately the leaders of the MIA and HA. The heads of the information department in HA were also consulted in B and C. The content of the interview was the same as the above. The specific timeframe for each interview was up to the interviewees and varied from 45 min to 90 min.

Given that hospitals are core stakeholders, we also interviewed persons in charge of health informatics from five well-known hospitals in Beijing. As the capital of China, Beijing has a large concentration of leading hospitals. We visited five hospitals in succession and consulted them on the status of their EMR systems, in addition to their data collection policies, usage, storage and level of sharing. Meanwhile, we canvassed their opinions on data integration and suggestions they would make to MIA and HA in this regard. The length of the interview was the same as the above.

Additionally, two group interviews were held with representatives of related companies. EMR data integration was mainly outsourced to IT companies, which are the actual data handlers. Ten interviewees from two MI information technology enterprises and five health information technology enterprises expressed their opinions. Moreover, we also met with representatives of two commercial health insurance companies (4 interviewees) as a supplementary survey of EMR data commercialization. The two group interviews were both about 2 h. The subjects covered were largely the same as the above. The discussions focused on the authorization, openness and commercialization prospects of EMR data integration.

## Findings

Data sharing and openness provide valuable results for ongoing patient care, and encourage innovation in public and private health institutions [19]. Although stakeholders are aware of the potential rewards, China’s EMR data integration progresses slowly. This lack of development puzzles many and our research aims to shed some light on the problem. Through interviews, we discovered the current situation of data segmentation, the mechanisms employed by MIA and HA, and the bottlenecks to data integration.

### Data segmentation of three parties

As can be seen from Fig. 1, there are three distinct formats when EMR data has been collected and compared: data from hospitals, MIIS and RHIP. Each have their own characteristics and differences.

1. Data from hospitals

The generation of EMR data arises from the process of routine clinical treatment, and includes the records of outpatient and emergency visits, hospitalization, medical imaging and so on. However, the EMR data of a hospital is normally confined to this hospital alone – there is no interaction with data from other hospitals. Moreover, independent and uncoordinated EMR systems in hospitals lead to heterogeneity problems and challenges in data integration.

(2) Data from MIIS

Data from MIIS integrates the EMR data of the insured person from all designated hospitals, using the personal identification number as the key. However, it doesn’t carry the data for non-insured persons or non-designated hospitals, nor the medicines or services provided, unless they are on the reimbursement list. In addition, MIIS is mainly concerned with economic indicators, thus medical imaging data is not collected.

(3) Data from RHIP

RHIP integrates a variety of data from hospitals administered by the local government. As we know, China’s population is highly mobile. Many people seek medical services across the provinces. Consequently, RHIP doesn’t have the data on local residents who see a doctor outside their area. As an important function of RHIP is to serve local residents, this missing data will have a significant negative impact on offering well-informed medical care.

### Mechanisms of MIA and HA

In order to obtain data efficiently, both MIA and HA exert influence on hospitals in the construction of information systems relating to EMR. For example, they will propose the standards of data format, storage, processing and so on. The different administrative functions and data requirements of both parties, determine the different administrative measures applied, as shown in Table 2.

**Table 2:** Data mechanisms of the two administrations

Administrations	MIA	HA
Data subjects	Patients registered with MI.	Local residency is essential. Some RHIP’s data cover all the patients of the local hospitals.
Data format standards	Urban population data have a unified format that hospitals must follow. Rural population data are gradually incorporated into urban population data with the basic result of a unified format.	There are big differences between provinces. Some provinces have proposed uniform standards, such as Jiangsu. However, in most of the provinces, prefectural standards are proposed. Some counties have their own standards. The hospitals mainly set the data format according to the requirements of the competent administration.
Data content	The system mainly collects all economic data of registered patients in real time. A holistic medical history is not recorded. Outpatient medical records include the medical record home page, medical record, prescription and billing. Real-time inpatient medical records include the inpatient medical record home page, admission records, surgical consent, anaesthesia consent, blood transfusion informed consent, critically ill (heavy) notice, prescription and billing, etc.	Most of the RHIPs regularly collect all kinds of medical record data, daily or weekly. Some of them don’t collect economic data such as price and total cost of drugs. Outpatient medical records include the medical record home page, medical record, laboratory reports, medical imaging data and so on. Inpatient medical records include the inpatient medical record home page, admission records, disease record, surgical consent, anesthesia consent, blood transfusion informed consent, special examination (special treatment) consent, critically ill (heavy) notice, medical order, auxiliary examination report form, body temperature list, medical imaging report, pathology report, etc.
Data quality	Data quality is stable and reliable. Eligibility and quotas of MI reimbursement are the key indicators which affect hospitals’ behaviour.	The data quality varies. There are no effective quality control measures.

Hospitals are administered by HA, but their economic lifeline is mainly controlled by MIA. More than 50% of the revenue of most hospitals comes from MIA. MIA has effective incentive mechanisms, because it can decide the eligibility and quotas of MI reimbursement. As for HA, the incentive mechanism is the financial allocation for equipment purchase and hospital expansion, which accounts for less than 20% of revenue of hospitals. This explains why the data quality of MIA is better than that of HA.

### Bottlenecks of integrating EMR data

EMR data is very difficult to share or release publically because of privacy and confidentiality concerns [20]. However, in order to explore the potential value of EMR data, many countries are making breakthroughs in this field. At this stage, poor data integration hinders the realization of the government’s goals in China. The key challenges of integration are as follows.

1. Authorization to EMR data collection

Recently, most hospitals in China have adopted the EMR system. Patients’ data is collected during their attendance at hospital. In the United Kingdom or other developed countries, there is an agreement that the patient can choose whether or not to share his/her medical data at the doctor’s visit. However, almost all Chinese hospitals do not have privacy agreements or personal data contracts with patients when collecting their data. In other words, the use of EMR data is not explicitly approved by the patient.

What’s more, there is no legal explanation and definition of the ownership of EMR data. The relevant laws about data security and confidentiality are as follows:

Article 6 of the Regulations on the Management of Medical Records of Medical Institutions: Medical institutions and their medical personnel shall strictly protect the privacy of patients and prohibit the leakage of medical records of patients for non-medical, teaching and research purposes.

Article 8 of the Management Regulations on Application of Electronic Medical Record (Trial): The terms, codes, templates and data used in electronic medical records shall comply with the requirements of relevant industry standards and norms, and promote the effective sharing of electronic medical record information under the premise of ensuring information security.

Article 6 of the Measures for the Management of Population Health Information (Trial): Responsible institutions should comply with the provisions of laws and regulations, follow medical ethical principles, ensure information security and protect personal privacy when collecting, utilizing and managing population health information. Whether the EMR data belongs to the patient or the hospital in practice is always legally disputed. The prevailing practice treats patient medical records as physical property that is owned by physicians and hospitals, and it allows patients and insurers to have access to the records [21]. However, the law does not grant providers exclusive ownership of healthcare records, which can be readily transferred [22].

At present, business interests and privacy concerns raised from big data analytics drive key stakeholders trying to resolve or change the ownership issue of EMR. Since many parties covet access to this enormously valuable data, legal risks are everywhere. Therefore, hospitals are very cautious and conservative about the handling and sharing of these data and wish at all costs to avoid unnecessary trouble. The blurred ownership of healthcare data blocks its authorized use and poses a huge hazard to the integration of EMR data.

(2) Uniformity of information standards

The non-uniform standard of medical data collection is a major factor blocking its integration in China, thus hindering the development of healthcare big data analysis [23]. In some fields, China has developed its own national standards, which it has learned from international norms and practice. This includes an adapted version of ICD-10, which is now used widely for reporting epidemiological data in China. SNOMED CT is being promoted, but its progress is painfully slow and difficult, due to the characteristics of the Chinese language. In general, the level of the formulation and implementation of standards are uneven between provinces or ministries.

MIA has made some progress in the standard setting. The standard Classification and Codes for Medications Covered by Social Insurance (LD/T90–2012) was released in 2012, and the standard Classification and Codes of Medical Services Covered by Social Insurance (LD/T01–2017) was released in 2017. With the aid of these standards, exchange and sharing of national medical insurance data can be conducted. They will also play an important supporting role in the delicate management of MI for cross-provincial patients, such as direct settlement, monitoring of medical services and payment standards.

HA is also advancing work in this field. Management Regulations on Application of Electronic Medical Record (Trial) was issued in February 2017. Standards and Regulations on Hospital Informationization Construction (Trial) was issued in April 2018. However, the construction of an information standard for RHIP still has a long way to go, including issues such as the overall design of an information platform, the data standards of the EMR, the standard for Picture Archiving and Communication Systems.

Pivotal non-uniform standards are as follows:

- First, independent and uncoordinated purchase of EMR systems leads to repeat purchases and inconsistent standards. EMR system providers set their own information standards. Public hospitals functioning independently, frequently buy EMR systems from different providers. Which leads to multiple systems in use in just one district, not to mention the waste of funds incurred.
- Secondly, healthcare classification and coding are not uniform. Most hospitals produce their own disease codes, billing codes, drugs, herbs and supplements databases. A few provinces develop their own province-wide standards. China has its own drug classification standard. This classification standard is not efficient due to the inclusion of many Chinese traditional medicines. ATC is used by only a few large hospitals. So, multiple versions of healthcare classification and coding standards militate against effective data integration. Such diversity is also a waste of public finance.

(3) Hospitals’ willingness to share data

As the primary EMR data controller, hospitals often have several disincentives for sharing the data they have collected. Inevitably it increases costs and may require the development of new information systems. Hospitals would need to categorize the data to determine what can be shared, adjust its format, provide access to a suitable interface, and even employ extra staff to take responsibility for these tasks.

Furthermore, sharing EMR data may also reduce hospital revenue. Where data is not shared, patients will pay the hospital for a range of diagnostic tests, even if similar tests might have already been carried out in previous hospitals. Effective data sharing between hospitals would obviate the need for multiple repeat tests and reduce their income accordingly.

In addition, hospitals are concerned about the social risks of data sharing. For example, misdiagnosis exists in any hospital. Once the data is shared or open, some misdiagnosis cases will inevitably be found, which may lead to claims and disputes which the hospitals wish to avoid.

Overall, the development of discrete health information silos in multiple hospitals, not only results in duplicate patient data and waste of medical resources, but also hampers the systematic development and construction of healthcare big data [24].

(4) Frictions caused by administrative decentralization

The decentralization of MIA and HA was designed to control healthcare costs. After several years of development, this has basically been achieved. To start with, the number of designated hospitals is increasing, so that insured patients now have more choices for their healthcare, and the level of medical treatment has been improved as well. Moreover, the expanding funding pool has emboldened the MIA to negotiate vigorously with hospitals and pharmaceutical companies. In response, the pharmaceutical companies have taken the initiative to lower their prices to gain entry onto the MIA reimbursement drug list, resulting in smaller margins per item but achieving quick turnover, so that both pharmaceutical company and patients’ interests are gradually balanced.

Unexpectedly, this mechanism design is not conducive to data integration. This is because both MIA and HA are in control of partial and different data resources (see Table 2), which they tend to guard in a partisan and exclusive manner. When this is coupled with years of conflict between them, it is not difficult to see why working together towards data consolidation is fraught and problematic.

(5) Public finance investment

Although RHIPs have been in existence for several years, their development is still fairly experimental and a mature and established structure is yet to emerge. They rely on public financial support since local governments are not willing to accept private capital. Given the sensitivity and confidentiality of patient data, local governments are concerned that profit-seeking private capital would inevitably bring risks if data sharing was opened up. The varying levels of financial investment available in different regions of China have dictated the rate of development of RHIPs. Jiangsu, a financially strong province, has basically completed the construction of provincial- prefectural -county RHIPs and is at the forefront of the country. For provinces with financial constraints, provincial platform constructions are facing great challenges, let alone at the prefectural and county RHIP level.

## Conclusion and recommendations

There is a recognised value and gradual trend towards data sharing and openness in the field of healthcare. Based on the above discussion regarding segmentation, incentive mechanisms and bottlenecks of EMR data integration, the establishment of a national integration and data sharing platform will necessarily involve various regulators and participants. Innovative institutional arrangements will be needed to raise the enthusiasm and support of all parties during the construction, development and trialing of such a platform. In response to the above bottlenecks, the following suggestions are proposed.

### Adopt a protocol for multi-stakeholder participation of data collection

Chapter 4 of the Cybersecurity Law of the People’s Republic of China regulates personal information protection and legally establishes several basic data protection principles, which are in accordance with international data protection measures. With this law in mind, a multi-stakeholder agreement should be reached as soon as possible, in order to balance the interests of all parties.

First of all, to ensure Individual Control and Transparency of patient data, patients should be clearly informed about the benefits and risks at stake in the handling of their data and its possible use more widely. Patients can then decide whether their EMRs should be collected and made available for large scale data projects. After the initial decision is made, patients maintain the right to change their minds without any difficulty. At present, patients have no control over the usage of their data and their wishes are not taken into account at all. This situation must be changed as soon as possible, otherwise, data collection will be boycotted or criticized by patients.

Secondly, the right of hospitals to control, use and benefit from patient medical data should be acknowledged, and their responsibility for privacy protection and data accuracy of patient records should be affirmed as well. Hospitals need to invest in higher quality data production and its proper oversight and maintenance. With the responsibility of protecting privacy and guaranteeing data accuracy, granting a hospital the rights to use patient data and benefit from the advantages that brings, should encourage it to better protect and value accuracy in its development [25].

Thirdly, the government should retain the right to use the data for the public interest. Hospitals should report key patient data in anonymized and de-identified forms to public authorities, which will create aggregate databases to promote public health, patient safety, and research [26].

### Co-establishment of information standards

In order to fully realize the value of the data, the existing standard system of MIIS and RHIP is far from being sufficient. More importantly, MIA and HA should work together on information standards.

For a start, a standardized and interoperable EMR should be implemented throughout the country. Such EMRs have been promoted and accepted in many countries. For example, in 2015 Switzerland passed a federal law that requires hospitals to adopt interoperable EMRs to facilitate data sharing and cooperation among healthcare providers [27]. MIA has a relatively better foundation, and a common EMR template can be developed based on it. Since patients are the beneficiaries, the earlier the hospital adopts the template, the more it will be welcomed by patients. Coupled with the government’s promotion, the popularity of the template should not take too long to achieve.

Secondly, healthcare coding should be standardized. For example, in the case of drugs not included in the reimbursement list of MIA, different codes are adopted in different hospitals. They are difficult to identify, thus their effect cannot be evaluated comprehensively. Therefore, the two administrations should cooperate to establish a national standard system by rationalising and integrating many provincial and prefectural standards, as well as the introduction and reference to international standards.

### Co-establishment of data audit mechanism

The MIA can obtain high-quality data from hospitals because of its good control and real-time financial settlement system. When it comes to the HA, some hospitals are often selective or even perfunctory in providing data. Thus, a clear data audit mechanism should be established.

Auditing the quality of the collected EMR data is a required first step. EMR data are produced by many hospitals, but the differences between doctors and hospitals inevitably mean there are considerable quality fluctuations in the material acquired. Rewarding or penalising hospitals according to the results of the audit would be a mechanism for bringing all providers to a uniformly required standard.

Next, privacy audits should be carried out. As a form of information assets, the protection of EMR data can be divided into two categories. The raw EMR data should be protected from the perspective of personal rights as they contain large amounts of private information [28]. Secondly, the processed data should be protected within the framework of intellectual property or business secrets, provided they have passed the privacy audits. Data products that have cleared the threshold of the privacy audits can benefit hospitals or providers since they have intellectual and economic value. What’s more, they can promote innovation and entrepreneurship.

### Expanding data integration for agglomerative effectiveness

Private ownership of EMR precludes forming comprehensive databases required for many important public health and safety applications. It also leads to the problem of data monopolies that will limit competition in the market for derived services [26]. Data integration should not be limited to MIIS and RHIP. It might be further explored and expanded in education, science and technology, public security, civil affairs, and human resources, in order to achieve effectiveness from scale.

First of all, there should be a strong push from both central and local governments. Many local governments in China are exploring ways to improve public services through data integration. As an example, significant progress has been made in Beijing, using its “Beijing Civil Social Service Card”(BCSSC). It can be used for many functions such as financial transactions, public transport, medical care, old-age pension payments and so on, and it will gradually integrate existing social security cards, hospital cards and traffic cards into one card. Project supervisors continue to monitor the progress of the BCSSC’s implementation, which to the end of 2016 saw the distribution of 12,259,000 cards.

Meanwhile, data sharing also needs to take into account the interests of the actual controllers in order to motivate their enthusiasm for engagement and participation. As of 2017, the BCSSC had integrated the EMR of 30 hospitals. Before this data consolidation took place, the health administration funded these hospitals, including data standards and system development.

### Adopting the public-private partnerships model

The Public-Private Partnerships Model is an innovation of cooperation between government and social capital, which can integrate different kinds of social resources, and effectively reduce the burden of government investment [29]. Using this model could promote the early realization of the policy goals, and also improve the economic and social benefits of big data development and application.

However, with the entry of private capital into this field, patient privacy concerns should be addressed appropriately beforehand. The entry of private capital is inevitable. In the light of this situation, patient concerns about the protection of their confidentiality will inevitably increase. To solve this problem, necessary adjustments about patient consent for the use of their medical data can be offered. Models such as Opt-Out, Broad/Blanket consent, Dynamic consent, and Meta consent [15] can be considered and adapted as needed.

In addition, there is a need for a sustainable business model. The platform can provide some value-added services. For individuals, it might provide health management, health consultation, and chronic disease management through memberships or customized value-added service fees. For pharmaceutical companies, it is able to provide marketing consultations for consulting fees or help them to advertise accurately according to the data mining of drug sales. For insurance companies, corresponding data services or special databases are welcome, which are the basis of their product development.
